# Comparing risk factors in severe COVID-19 using machine learning and non-machine learning methods: analysis from 2 international randomized controlled trials

**DOI:** 10.1093/jamiaopen/ooag079

**Published:** 2026-06-23

**Authors:** Christian Møller Jensen, Ramtin Zargari Marandi, Kasper Sommerlund Moestrup, Ahmad Mourad, Alfredo J Mena Lora, Brad T Sherman, David M Vock, Jacqueline A Nordwall, Joanne M Carson, Nathan Peiffer-Smadja, Neil R Aggarwal, Nicole E Naiman, Samuel M Brown, Thomas W Barrett, Timothy Hatlen, Victoria S Kjærgaard, Weizhong Chang, Matthew R Sydes, Jens Lundgren, Tomas O Jensen, for the STRIVE Network and ITAC and TICO Study Groups, Mark N Polizzotto, Jacqueline Nordwall, Abdel G Babiker, Andrew Phillips, David M Vock, Nnakelu Eriobu, Vivian Kwaghe, Roger Paredes, Lourdes Mateu, Srikanth Ramachandruni, Rajeev Narang, Mamta K Jain, Susana M Lazarte, Jason V Baker, Anne E P Frosch, Garyfallia Poulakou, Konstantinos N Syrigos, Gretchen S Arnoczy, Natalie A McBride, Philip A Robinson, Farjad Sarafian, Sanjay Bhagani, Hassan S Taha, Thomas Benfield, Sean T H Liu, Anastasia Antoniadou, Jens Ulrik Stæhr Jensen, Ioannis Kalomenidis, Adityo Susilo, Prasetyo Hariadi, Tomas O Jensen, Jose Luis Morales-Rull, Marie Helleberg, Sreenath Meegada, Isik S Johansen, Daniel Canario, Eduardo Fernández-Cruz, Simeon Metallidis, Amish Shah, Aki Sakurai, Nikolaos G Koulouris, Robin Trotman, Amy C Weintrob, Daria Podlekareva, Usman Hadi, Kathryn M Lloyd, Birgit Thorup Røge, Sho Saito, Kelly Sweerus, Jakob J Malin, Christoph Lübbert, Jose Muñoz, Matthew J Cummings, Marcelo H Losso, Dan Turner, Kathryn Shaw-Saliba, Robin Dewar, Helene Highbarger, Perrine Lallemand, Tauseef Rehman, Norman Gerry, Dona Arlinda, Christina C Chang, Birgit Grund, Michael R Holbrook, Horace P Holley, Fleur Hudson, Laura A McNay, Daniel D Murray, Sarah L Pett, Megan Shaughnessy, Mary C Smolskis, Giota Touloumi, Mary E Wright, Mittie K Doyle, Sharon Popik, Christine Hall, Roshan Ramanathan, Huyen Cao, Elsa Mondou, Todd Willis, Joseph V Thakuria, Leman Yel, Elizabeth Higgs, Virginia L Kan, Jens D Lundgren, James D Neaton, H Clifford Lane

**Affiliations:** CHIP, Centre of Excellence for Health, Immunity, and Infections, Rigshospitalet, University of Copenhagen, Copenhagen Ø, 2100, Denmark; CHIP, Centre of Excellence for Health, Immunity, and Infections, Rigshospitalet, University of Copenhagen, Copenhagen Ø, 2100, Denmark; CHIP, Centre of Excellence for Health, Immunity, and Infections, Rigshospitalet, University of Copenhagen, Copenhagen Ø, 2100, Denmark; Division of Infectious Diseases, Department of Medicine, Duke University School of Medicine, Durham, NC 27710, United States; Duke Clinical Research Institute, Durham, NC 27701, United States; University of Illinois Chicago, Chicago, IL 60607, United States; Frederick National Laboratory for Cancer Research, Frederick, MD 21701, United States; Division of Biostatistics & Health Data Science, University of Minnesota, Minneapolis, MN 55414, United States; Division of Biostatistics & Health Data Science, University of Minnesota, Minneapolis, MN 55414, United States; Kirby Institute, University of New South Wales, Sydney, NSW 2052, Australia; Université Paris Cité et Université Sorbonne Paris Nord, Inserm, IAME, Paris, 75018, France; Infectious and Tropical Diseases Department, Hopital Bichat – Claude Bernard, AP-HP, Paris, 75018, France; Division of Pulmonary, Allergy, and Critical Care Medicine, University of Colorado School of Medicine, Aurora, CO 80045, United States; Department of Internal Medicine, University of Texas Southwestern Medical Center, Dallas, TX 75390, United States; Pulmonary/Critical Care Medicine, Intermountain Medical Center, Salt Lake City, UT 84132, United States; VA Portland Health Care System, Portland, OR 97239, United States; Oregon Health & Science University, Portland, OR 97239, United States; Harbor-UCLA Medical Center, Torrance, CA 90502, United States; CHIP, Centre of Excellence for Health, Immunity, and Infections, Rigshospitalet, University of Copenhagen, Copenhagen Ø, 2100, Denmark; Frederick National Laboratory for Cancer Research, Frederick, MD 21701, United States; MRC Clinical Trials Unit at UCL, Institute of Clinical Trials and Methodology, UCL, London WC1V 6LJ, United Kingdom; Data for R&D, Transformation Directorate, NHS England, London, United Kingdom; CHIP, Centre of Excellence for Health, Immunity, and Infections, Rigshospitalet, University of Copenhagen, Copenhagen Ø, 2100, Denmark; Department of Infectious Diseases, Rigshospitalet, University of Copenhagen, Copenhagen Ø, 2100, Denmark; Department of Clinical Medicine, University of Copenhagen, Copenhagen N, 2200, Denmark; CHIP, Centre of Excellence for Health, Immunity, and Infections, Rigshospitalet, University of Copenhagen, Copenhagen Ø, 2100, Denmark

**Keywords:** SARS-CoV-2, randomized controlled trial, machine learning, medical informatics, biostatistics

## Abstract

**Objective:**

To compare differences in risk factors and 90-day mortality prediction from 2 machine learning (ML) models with a previously published non-ML model and investigate their validity in an external cohort.

**Materials and methods:**

Prospectively collected data from 2 separate randomized controlled trial (RCT) cohorts from 2020 to 2021, the Therapeutics for Inpatients with COVID-19 (TICO/ACTIV-3) Trial (derivation and internal validation cohort) and the Inpatient Treatment with Anti-Coronavirus Immunoglobulin (ITAC) Trial (external validation cohort) were used. Data were collected from 114 sites in 10 countries (TICO/ACTIV-3) and 63 sites in 11 countries (ITAC). A ML pipeline including 5 classification models, and 1 survival model was used for risk factor identification and clinical outcome prediction. Risk factors were compared between a ML-based classification model, a ML-based survival model and a previously published Cox model. Performance of the ML-based classification model was compared across TICO/ACTIV-3 and ITAC.

**Results:**

A total of 2625 (TICO/ACTIV-3) and 579 (ITAC) adults hospitalized for COVID-19 were included. Some overlap of risk factors was identified across models. Five were identified in all models, 3 only in ML models, and 4 only in the non-ML model. The ML model showed good predictive performance in TICO/ACTIV-3. Internal validation showed no overfitting. Lower model performance was observed in ITAC (−15.8%), but performance remained above chance level.

**Discussion:**

Differences in methods for risk factor identification using ML and non-ML complicates the comparison of results derived from each approach, but using multiple approaches may unveil overlooked risk factors.

**Conclusion:**

Risk factor identification may benefit from integrating both ML and non-ML methods, but external validation is necessary, even in RCTs.

## Background and significance

Secondary analyses of the Therapeutics for Inpatients with COVID-19 (TICO/ACTIV-3) platform trial cohort have identified risk factors for adverse outcomes in patients hospitalized for coronavirus disease 2019 (COVID-19), mainly through non-machine learning (ML) models.^1^^,^^2^

In contrast, ML models have been used to explore risk factors for adverse outcomes using smaller datasets from the initial COVID-19 wave in 2020; however, these studies were mainly done using retrospectively collected data or nonheterogenous populations.^3–8^

The use of ML, a branch of artificial intelligence focused on creating algorithms that learn by finding patterns in large datasets, has become increasingly popular in the medical literature over the last decade.^9^^,^^10^ Application of ML algorithms using prospectively collected data from large-scale international trials has been described previously in conditions other than COVID-19.^11^^,^^12^ ML models present potential significant advantages on some parameters compared to non-ML models, particularly when uncovering complex, non-linear relationships and interactions among features, but studies directly comparing model performance and risk factor identification between ML models and non-ML models are scarce. However, in 1 large prospective observational study, a supervised ML method known as Random Survival Forests (RSF) outperformed prediction models based on non-ML methods, improving prediction accuracy by 10%-25%.^13^

An important criticism of ML-based prediction models is the risk of overfitting and the resulting limited generalizability to other datasets. Improved predictions in ML models may come at the cost of poor model generalizability when applied to other studies or in practice, and methods frequently used in ML to mitigate the risk of overfitting, may not always lead to increased model stability.^14^^,^^15^ To secure generalizability, external validation of developed prediction models is recommended.^16^

## Objective

We used ML models to identify risk factors of 90-day mortality amongst hospitalized patients with COVID-19 in TICO/ACTIV-3 and compared results with a previously published non-ML analysis on the same cohort. We further aimed to validate findings using a separate but comparable cohort from the Inpatient Treatment with Anti-Coronavirus Immunoglobulin (ITAC) trial.^17^

## Methods and materials

### Study design and data collection

This study was an exploratory, retrospective study using prospectively collected data from the TICO/ACTIV-3 platform trial and the ITAC trial.^1^^,^^17^ Further information on the original study protocols and their eligibility criteria can be found in their respective protocols. The study was conducted in accordance with the Transparent Reporting of a multivariable prediction model for Individual Prognosis Or Diagnosis—Artificial Intelligence (TRIPOD-AI) statement.^18^

### Participants

To align with the non-ML analysis of TICO/ACTIV-3, we included all participants in the modified intention-to-treat analysis randomized between August 2020 and November 2021 to placebo, 1 of 4 neutralizing monoclonal antibodies, or a Designed Ankyrin Repeat Protein.^2^ Day-90 all-cause mortality was used as a secondary outcome measure, and only 1 of the agents (tixagevimab–cilgavimab) was associated with lower mortality when compared with placebo.^19^

All participants included in the mITT analysis of ITAC comprised the external validation cohort.^17^ Participants were randomized between October 2020 and February 2021 to placebo or hyperimmune intravenous immunoglobulin (hIVIG). All-cause mortality through day 28 was a secondary outcome measure, and hIVIG failed to affect clinical outcomes when compared with placebo.^17^

### Data

TICO/ACTIV-3 and ITAC were independent studies with distinct populations, with a considerable overlap between the baseline features collected in both studies.^1^^,^^17^ Features available in both cohorts were included in the ML analyses. Additional composite comorbidity features were derived from individual comorbidity features and included in the ML models. All features were quality checked manually, and missing data were imputed using the k-Nearest Neighbor algorithm.

### Outcomes

All-cause mortality was first assessed through day 90 in TICO and then through day 28 in both TICO and ITAC. All patients lost to follow-up (LTFU) prior to binary outcome measure times were defined as having no event (alive) and were included in the analyses.

### Machine learning analyses

An 80/20 random data split into training and test sets was performed with stratification to preserve the proportion of outcome events in each set. We used the Medical Artificial Intelligence Toolbox (MAIT) as a ML pipeline implemented in Python for the ML analysis, particularly in the prediction of 2 types of outcomes, namely, a binary outcome variable using binary classification models and a time-to-event outcome variable using a survival model.^20^ From MAIT, we included 5 binary classification models, Random Forest,^21^ Light Gradient-Boosting Machine (LightGBM),^22^ CatBoost,^23^ Histogram-based Gradient Boosting Classification Tree (HistGBC),^24^ and Logistic Regression regularized on L1.^25^ The following performance measures were derived and reported for each binary classification model: positive predictive value (PPV), negative predictive value (NPV), sensitivity, specificity, balanced accuracy ((sensitivity + specificity)/2), Matthews correlation coefficient (MCC), receiver operating characteristic area under the curve (ROCAUC), precision-recall area under the curve (PRAUC), and the Brier score. 5-fold cross validation was used to derive standard deviations in the training set, and bootstrapping was applied to the test set and external validation set to estimate 95% confidence intervals for each performance metric. The average of MCC, ROCAUC, and PRAUC was estimated and denoted as “MRPAvg.” MRPAvg was used as a composite measure to determine the best performing model alongside a criterion of Brier score <0.2. These metrics were selected to provide a comprehensive evaluation of binary classification models, addressing both discrimination and calibration. ROCAUC and PRAUC assess the overall model’s ability to distinguish between positive and negative cases, especially in imbalanced datasets. Brier score reflects model calibration while MCC and balanced accuracy provide balanced assessments of overall model accuracy.

To account for time-to-event and censoring, 1 survival-based model, RSF, was applied as a sensitivity analysis, and the predicted cumulative hazard was estimated.^26^ The concordance index and integrated Brier score were reported as survival model-appropriate performance metrics. A transformation of the predicted cumulative hazard as the output of the RSF model into binary classes was done based on Euclidean distance of individual predicted cumulative hazards from the test set with the median of the predicted cumulative hazard for each class from the training set. Binary class performance measures (NPV, PPV, sensitivity, specificity, balanced accuracy, and MCC) were derived post-transformation.

We performed hyperparameter tuning for all algorithms using random search within a predefined parameter search space for each model. We used nested cross-validation models to ensure unbiased performance estimates during hyperparameter tuning. To identify the optimal parameters for each model, we employed 5-fold cross-validation repeated 10 times on the training set.

### SHapley Additive exPlanations (SHAP) analysis

SHAP values were extracted using the *shap* package for binary classification and the *survSHAP(t)* package for the RSF model.^27^^,^^28^ The SHAP values quantify the contribution of each feature to a model’s prediction for a specific sample (eg, a patient). The sum of SHAP values for all features, combined with the model’s baseline value, represents the model’s prediction for that sample. SHAP values quantify each feature’s contribution to a single prediction by attributing changes from the baseline (mean) prediction according to Shapley values from cooperative game theory. Positive SHAP indicates that the feature increases risk, and negative indicates a protective or lowering effect. A SHAP value of zero indicates no contribution from the feature to the model’s prediction for that specific sample.

### Ethical approvals, original study registrations, and informed consent

Information on ethical approvals and study registrations can be found in the respective original studies.^1^^,^^17^ Written informed consent was given for all trials and secondary analyses.

## Results

### Baseline characteristics

Of 2625 participants in the TICO/ACTIV-3 cohort, 179 (6.8%) died by day 28 after randomization and 261 (9.9%) died by day 90. In ITAC, 40 (6.9%) of the total 579 participants died by day 28. Twenty-seven (1.0%) and 1 participant (<1%) were LTFU by day 28 in TICO and ITAC, respectively, while 41 (1.5%) participants were LTFU by day 90 in TICO.

A total of 107 unique baseline features (91.5% of all features) were included across ITAC and TICO. Participant characteristics, features included and feature missingness can be found in Table S1 and Supplementary File 2.

### Identification of risk factors for 90-day all-cause mortality using machine learning algorithms

All models had higher NPV than PPV, and specificity than sensitivity (Table 1).

**Table 1.** Mean values ± standard deviation of the performance measures of the 5 models predicting 90-day mortality, from 5-fold cross validation in the training set. *n* = 2100, events = 209.

	RandomForest	LightGBM	CatBoost	HistGBC	LogisticRegression
**PPV**	0.46 ± 0.13	0.4 ± 0.06	0.38 ± 0.02	0.38 ± 0.03	0.3 ± 0.02
**NPV**	0.93 ± 0.02	0.95 ± 0.02	0.96 ± 0.01	0.95 ± 0.0	0.97 ± 0.01
**Sensitivity**	0.33 ± 0.22	0.56 ± 0.16	0.65 ± 0.07	0.58 ± 0.05	0.74 ± 0.05
**Specificity**	0.94 ± 0.06	0.9 ± 0.04	0.88 ± 0.02	0.89 ± 0.02	0.81 ± 0.01
**Balanced Accuracy**	0.64 ± 0.08	0.73 ± 0.06	0.77 ± 0.03	0.74 ± 0.02	0.77 ± 0.02
**MCC**	0.3 ± 0.1	0.39 ± 0.05	0.42 ± 0.03	0.39 ± 0.03	0.38 ± 0.03
**ROCAUC**	0.86 ± 0.02	0.85 ± 0.03	0.86 ± 0.02	0.84 ± 0.03	0.85 ± 0.02
**PRAUC**	0.45 ± 0.02	0.41 ± 0.03	0.46 ± 0.03	0.39 ± 0.03	0.47 ± 0.07
**Brier Score**	0.11 ± 0.03	0.1 ± 0.01	0.11 ± 0.0	0.1 ± 0.01	0.14 ± 0.01
**MRPAvg**	0.54	0.55	0.58	0.54	0.57

Abbreviations: PPV=Positive predictive value; NPV=Negative predictive value; MCC=Matthew’s correlation coefficient; ROCAUC=Receiver operating characteristics area under the curve; PRAUC=Precision-recall area under the curve.

$\mathrm{MRPAvg}\;=\;\frac{\mathrm{MCC}+\mathrm{ROCAUC}+\mathrm{PRAUC}}{3}$ ; a higher MRPAvg means better overall prediction.

The best performing model was CatBoost (MRPAvg 0.58). The CatBoost model was applied to the test set (*n* = 525, events = 52) for internal validation. (Table S2; Figure S1) The CatBoost model showed no sign of overfitting when applied to the test set, with comparable MCC (0.42 vs 0.48), ROCAUC (0.86 vs 0.91), PRAUC (0.46 vs 0.58), and MRPAvg (0.58 vs 0.66) in the training set and test set, respectively. All performance measures in the test set were higher than chance. The estimated ROCAUC from the CatBoost model (0.91) was similar to the time-dependent ROCAUC derived from the final Cox proportional hazards model (0.90) reported by Aggarwal and colleagues^2^ (Figure S2).

**Figure 1.**
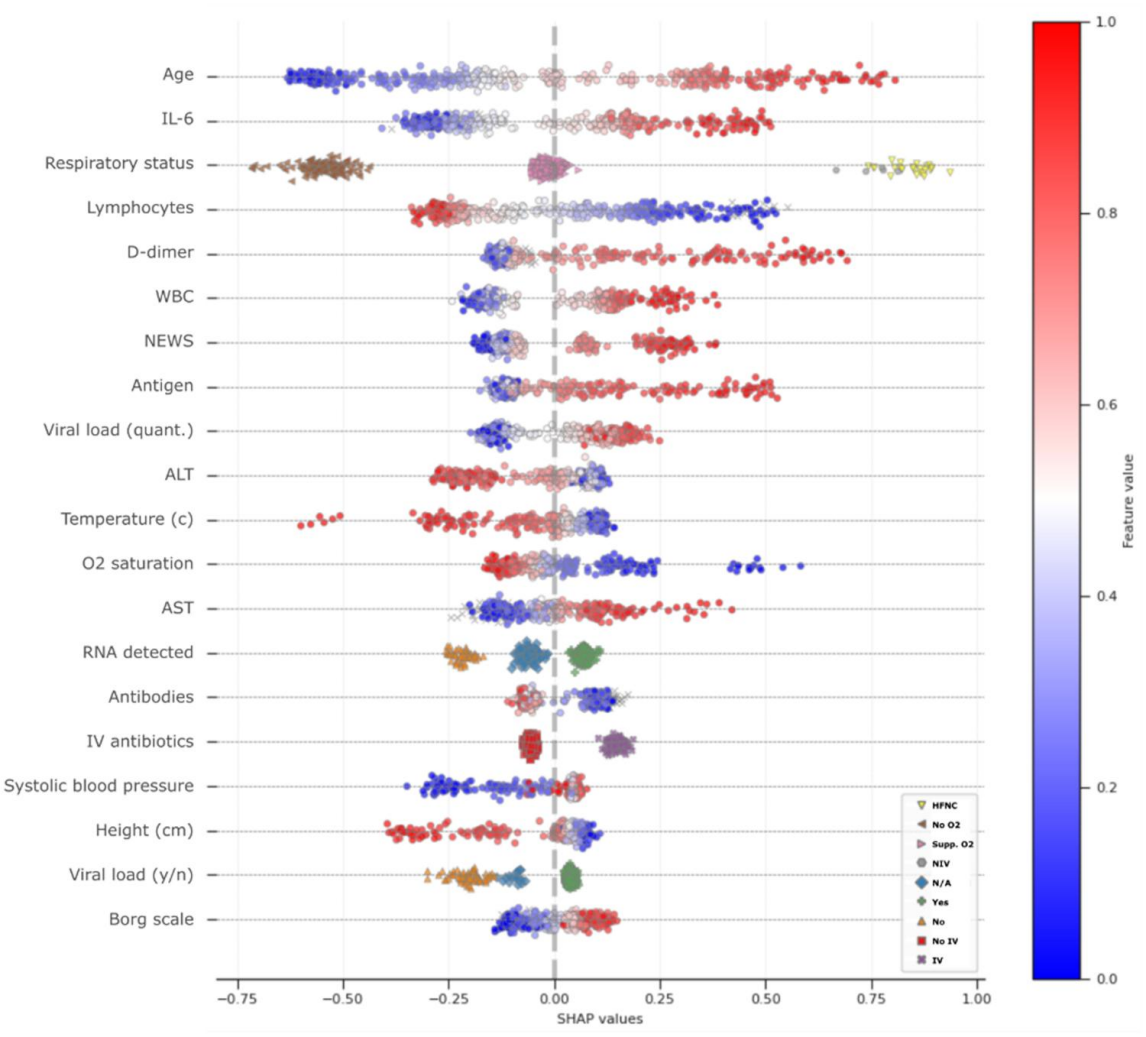
SHAP summary plot derived from the CatBoost model applied to the test set predicting 90-day mortality. Top 20 features are shown. Feature values of continuous features correspond to the blue-to-red scale on the right. Feature values of categorical features are explained in the figure legend (yes/no/not applicable/unknown for binary features, specific categories for the multilevel “Respiratory Support” feature). IL-6 = interleukin-6; WBC=white blood cell count; NEWS=National Early Warning Score; ALT=alanine transaminase; AST=aspartate transaminase; RNA=Ribonucleic Acid; IV=intravenous; HFNC=high-flow nasal cannula; Supp. O2 = supplemental oxygen; NIV=Noninvasive ventilation.

SHAP analysis was applied to the CatBoost model, and SHAP values for the top 20 features are shown for the overall test set, as well as for low-risk and high-risk clusters. (Figure 1; Supplementary Figures 3 and 4) Examples of SHAP force plots for individuals can be found in Figure S5.

**Figure 2.**
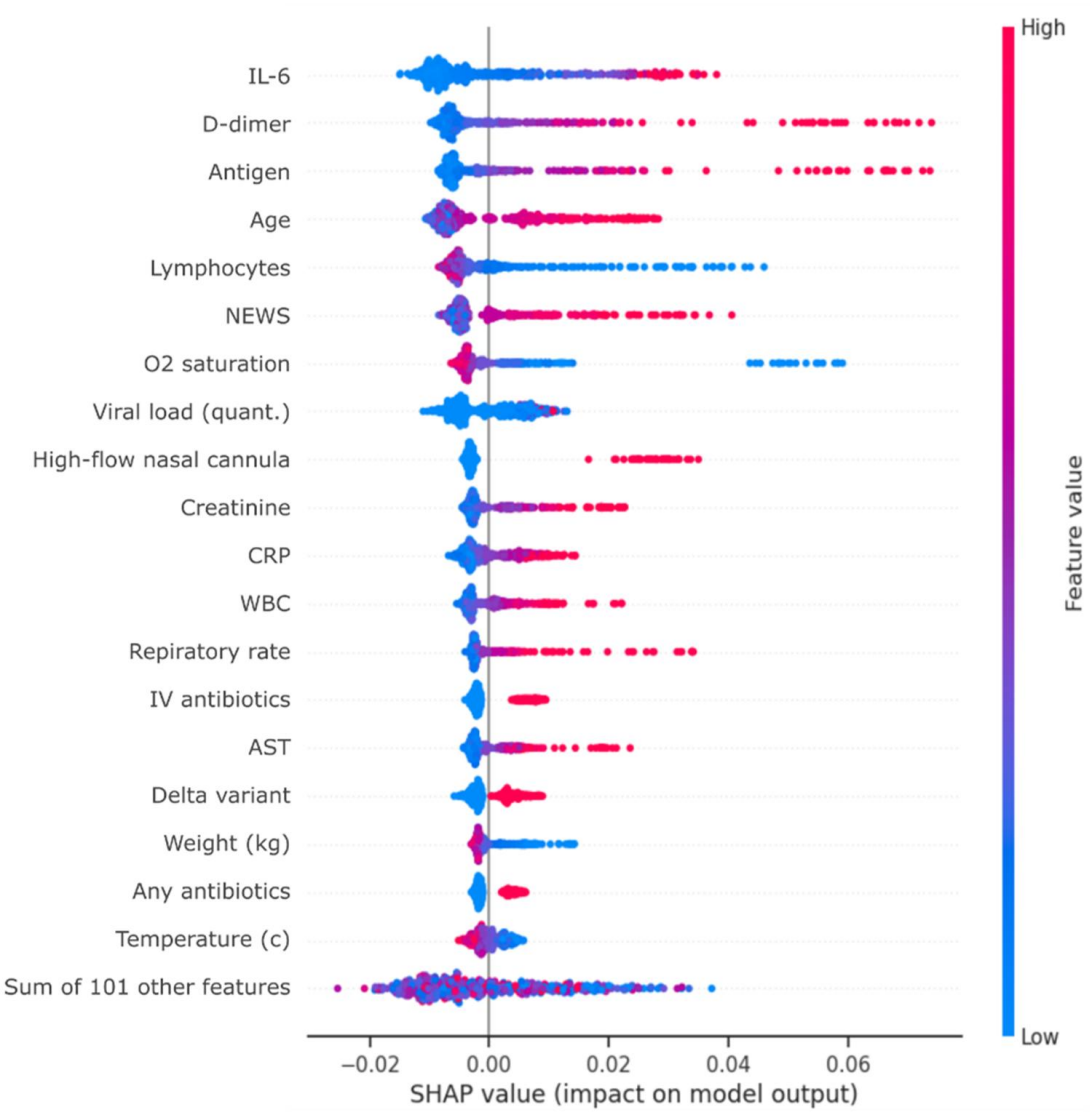
SHAP summary plot derived from the Random Survival Forests model applied to the test set predicting 90-day mortality. Top 20 features are shown. Each dot corresponds to the SHAP value of a respective feature for 1 patient. Feature values correspond to the blue-to-red scale on the right. For binary variables, red corresponds to the presence of a given feature (eg, a red dot in the “Delta variant” feature corresponds to being infected with a delta variant virus). Multi-level categorical variables were 1-hot-encoded and treated as binary features at each level (eg, HFNC is shown by itself instead of the respiratory status feature). IL-6 = interleukin-6; CRP=C-reactive protein; WBC=white blood cell count; NEWS=National Early Warning Score; ALT=alanine transaminase; AST=aspartate transaminase; IV=intravenous; BMI=body mass index.

RSF was then applied to the training and test sets to identify potential differences in risk factors between models based on binary classification (CatBoost) and time-to-event. The c-index and integrated Brier score were estimated for the training set with 5-fold cross validation (0.82 ± 0.03 and 0.06 ± 0.01, respectively) and the test set (0.87 and 0.06). The performance metrics showed no sign of overfitting. Next, the predicted cumulative hazard was estimated for the test set and translated to binary classes for comparability. (Figure S6; Table S3) SHAP values for all features were derived for the overall test set (Figure 2).

The 9 statistically significant features from the final Cox model (non-ML) by Aggarwal and colleagues,^2^ as well as the top 10 features from the CatBoost and the RSF models are compared in Table 2.

**Table 2.** Top 10 SHAP value features derived from the CatBoost model, vs statistically significant variables from the non-ML model from study by Aggarwal et al,^2^ vs top 10 SHAP value features derived from the RSF model.

Feature name	CatBoost model	Cox (Non-ML) model	RSF model
**Age**	X	X	X
**IL-6**	X	X	X
**Respiratory status**	X	X	X^e^
**Lymphocyte count**	X		X
**D-dimer**	X		X
**White blood cell count**	X		
**NEWS**	X		X
**Viral nucleocapsid antigen plasma**	X	X^b^	X
**Upper respiratory viral RNA (Quantified viral load)^a^**	X	X	X
**Alanine transaminase**	X		
**African region**		X^c^	
**European region**		X^c^	
**Infection period pre-2021**		X	
**eGFR**		X^d^	
**Peripheral oxygen saturation**			X
**Creatinine**			X

Top 10 features and statistically significant features are denoted “X.”

a Determined from the same nasal swap in 2 independent laboratories.

b ≥4500 ng/L vs < 200 ng/L.

c Using United States of America as the reference group.

d <60 mL/min/1.73 m^2^ vs ≥60 mL/min/1.73 m^2^.

e Receiving respiratory support using high-flow nasal cannula.

Three features (lymphocyte count, D-dimer, and National Early Warning Score [NEWS]) were identified as risk factors for 90-day mortality in both the CatBoost model and the RSF model, but not the Cox model. Five features (age, interleukin-6 [IL-6], respiratory status, quantified viral antigen in plasma, and upper respiratory viral ribonucleic acid [RNA]) were identified as risk factors in all 3 models. The remaining 4 out of 9 features identified as statistically significant in the Cox model (African region, European region, infection prior to 2021 and estimated Glomerular Filtration Rate [eGFR]) were not identified as the top 10 risk factors in the CatBoost or RSF model. When including the top 20 features instead of top 10, none of the 4 remaining risk factors were identified by the ML models. However, although eGFR, a feature derived from creatinine values, was not identified as one of the top 10 risk factors in the ML models, creatinine was included in the top 10 of the RSF model.

### Assessment of generalizability in an external population

The TICO cohort was split into new training and test sets (*n* = 2100 and 525, respectively). The MAIT pipeline was reapplied to the new training set using 28-day mortality as the new outcome measure. (Table S4) The LightGBM model performed best on the training data (MRPAvg = 0.55). The LightGBM model was applied to the test data and showed no loss of performance (MCC 0.38 vs 0.42; ROCAUC 0.87 vs 0.89; PRAUC 0.39 vs 0.41 in the training set and test set, respectively) (Table S5; Figure S7).

The LightGBM model was applied to the ITAC cohort (Table 3). The model had lower performance across all 3 performance measures but remained above chance level.

**Table 3.** LightGBM model performance with standard deviation (SD) or 95% confidence intervals (CI), predicting 28-day mortality.

	TICO training set (SD)	TICO test set (95% CI)	ITAC (95% CI)
**MCC**	0.38 ± 0.09	0.42 (0.29-0.54)	0.31 (0.19-0.44)
**ROCAUC**	0.87 ± 0.03	0.89 (0.84-0.93)	0.83 (0.76-0.89)
**PRAUC**	0.39 ± 0.08	0.41 (0.25-0.55)	0.33 (0.20-0.46)
**Brier Score**	0.08 ± 0.01	0.07 (0.06-0.09)	0.10 (0.09-0.11)
**MRPAvg**	0.55	0.57	0.48

## Discussion

Among patients hospitalized for COVID-19, we found a moderate overlap of identified risk factors between a previously published Cox (non-ML) model, a ML-based binary classification model (CatBoost) and a ML-based survival model (RSF). The ML models identified additional risk factors that were not identified in the Cox model, and vice versa, despite being modeled on the same population. Despite the additional features, the model performance did not increase significantly when using a ML-based approach compared to a non-ML approach. While internal validation showed no sign of model performance loss, external validation showed decreased model performance, but the model performance remained above chance level.

Age, respiratory status, IL-6, quantified viral nucleocapsid antigen in plasma and upper respiratory viral RNA were the 5 risk factors identified across all 3 models. These features are mostly well-ascertained risk factors of adverse outcomes at the time of admission.^29–31^ The overlap emphasizes the robustness of these features as predictors of mortality at the time of admission.

NEWS, D-dimer, and lymphocyte count were found in the top 10 of both ML models, but not in the non-ML model, although D-dimer was not included in the analysis for the non-ML model. NEWS was significantly associated with 90-day mortality in the unadjusted, but not adjusted, analysis in an early Cox model, and was not included in the final Cox model.^2^ NEWS, a composite feature comprising information on vital signs, has been extensively investigated in patients hospitalized for COVID-19.^32–35^ The majority of the studies were performed on early COVID-19 cohorts before dexamethasone and antivirals became the standard of care, and study conclusions were ambiguous. Some studies advocated for on-admission use of NEWS for short-term mortality prediction (<48 h), and others advocated primarily for longitudinal use, to track changes in NEWS as an indicator of clinical deterioration. Interestingly, one study found the respiratory components of NEWS (supplemental oxygen, respiratory rate, and hypoxemia) to be the main drivers of mortality prediction within NEWS.^35^ These findings may partially explain the lack of statistical significance of NEWS in the Cox model, due to collinearity with the respiratory status feature, as NEWS was significantly associated with 90-day mortality only in the unadjusted Cox model. Methods, such as regularization, were included in MAIT to mitigate the risk of collinearity, which may explain NEWS being important in the ML models. Lymphopenia (lymphocyte count < 0.9 × 10^9^/L) was associated with 90-day mortality in an early Cox model.^2^ However, in the final Cox model, a cutoff of ≤1.5 × 10^9^/L was used and was not associated with 90-day mortality. An association between lymphopenia and COVID-19 progression has been reported,^36^^,^^37^ and in a systematic review and meta-analysis from 2020, lymphopenia was associated with several poor outcomes including mortality using a cutoff of ≤1.1 × 10^9^/L.^38^ Our analysis suggests that the lack of significant association with 90-day mortality in the Cox model may be explained by the cutoff used.

ML analyses can often be better at handling large feature sets relative to the number of observations compared to traditional statistical methods, reducing the need for arbitrary exclusion of potentially meaningful predictors. Although some researchers have advocated for using a less conservative approach than the 10-events-per-predictor in binary logistic regression,^39^ and Cox regression,^40^ approaches such as feature selection based on *P-*values from univariable analysis, feature selection based on previous studies, stepwise regression techniques or stratifying analyses based on domains, as done in the study by Aggarwal and colleagues,^2^ are often used to work around these limitations although they may introduce bias. The MAIT pipeline utilized methodologies such as tree-based modeling, regularization and bootstrapping to mitigate the risk of overfitting despite providing an events-per-predictor ratio of approximately 2:1 (209 events to 107 predictors in the training set). Additionally, identification of risk factors is inherently influenced by the assumptions and algorithms underlying each model. For instance, the Cox model is semiparametric, requiring no specification of the baseline hazard, but assumes log-linear effects of covariates on the hazard. Extensions (eg, spline terms) allow flexible modeling of continuous predictors without assuming strict linearity. RSF and CatBoost use non-parametric and non-linear approaches, respectively. This divergence in methodology can cause certain features to appear important in one model but less so in another, depending on how the models handle interactions, correlations, and the functional forms of the features. Risk factors identified by all models should therefore be considered the most robust and deserving of the highest attention.

To address the recent concern of lack of generalizability of ML prediction models from randomized controlled trials (RCTs),^14^ ITAC was used for external validation. The ITAC cohort comprised a population comparable to that of TICO, with the main differences being differences in the regional distribution of trial participants (78.4% and 42.8% of the trial population recruited in North America in TICO and ITAC, respectively) as well as the percentage of patients having received at least 1 SARS-CoV-2 vaccination (20.1% and 2.1% in TICO and ITAC, respectively). Despite comparable cohorts, model performance was lower in the external validation. However, when assessing ROCAUC, PRAUC or MCC, performance was above chance level. The original Cox model was not externally validated in a separate cohort. While not overfitted, the decrease in ML model performance by 15.8% (MRPAvg 0.57 vs 0.48 in the TICO test set and ITAC, respectively) further emphasizes recent studies’ findings of the importance of externally validating models from RCTs and the reporting of model stability.^14^^,^^15^

This study has several strengths. The data used in this study were from two independent, global, multicenter RCTs from overlapping time periods. Furthermore, as part of the MAIT pipeline, SHAP methodology was used to further enhance interpretation and understanding of ML outputs, demonstrated by the SHAP plots. While SHAP methodology is not perfectly comparable with the output of a Cox model, this explainable ML method provided an alternative that was easy to interpret and allowed us to bypass the black-box paradox of artificial intelligence.

There are some limitations to this study. Patients LTFU prior to day 90 were classified as not having an event in the binary classification models, potentially leading to misclassification bias. However, only 1.7% of patients in the TICO cohort were LTFU, and the RSF model, which uses right-censoring, found similar risk factors and had similar performance on the test set (Table S3). Additionally, due to limitations in follow-up time in the ITAC cohort, a secondary analysis using 28-day mortality was made to externally validate the findings from the 90-day mortality analysis. However, similar performance was reported in both models in the test set. Lastly, the original Cox model was derived from 100% of the TICO population, while our ML models were derived using only 80% of the population (training set). While this limits direct comparability, it highlights the lack of internal validation in the conventional non-ML approach.

## Conclusion

Our results support the use of ML methods for risk factor identification and emphasize the need for external validation. While our study highlights the challenges of directly comparing results derived from ML and non-ML models, it provides an example of how ML and non-ML models can be merged to explore potentially overlooked risk factors for further investigation.

## Supplementary Material

ooag079_Supplementary_Data

## Data Availability

Data from both studies can be obtained from http://insight.ccbr.umn.edu/

## Code availability

The underlying code for this study is available online in the PERSIMUNE MAIT repository and can be accessed via this link: https://github.com/PERSIMUNE/MAIT
