# Supplementary material for: Comparing risk factors in severe COVID-19 using machine learning and non-machine learning methods: analysis from 2 international randomized controlled trials
Source: JAMIA Open. 2026 Jun 23;9(3):ooag079. doi: 10.1093/jamiaopen/ooag079 (PMC13289610; doi:10.1093/jamiaopen/ooag079)
Supplement: ooag079_Supplementary_Data [file ooag079_supplementary_data.zip › SupplementaryFile2.pdf]

TICO and ITAC features

Feature	TICO or ITAC	Included?	Reason	Total per group
Seq ID	Both	No	Patient identifer	
**(PT) Participant demographics**				
Treatment assigned	Both	Yes		
Country name	Both	No	Composite variable made instead (Region)	
Treatment dosage	Both	Yes		3
**(SC) Screening**				
Sex	Both	Yes		
Age	Both	Yes		
Assigned site	Both	No	For internal use only	
Symptom duration (days)	Both	Yes		4
**(BR) Baseline and randomization**				
Ethnicity	Both	No	Low solution, did not allow for multiple ethnicities.	
Asian ethnicity	Both	Yes		
Black ethnicity	Both	Yes		
White ethnicity	Both	Yes		
Hispanic ethnicity	Both	Yes		
Other ethnicity	Both	Yes		
Participant's residence	TICO	No	Insufficienty data for validation	
Participant experience any of the following comorbidity	Both	Yes		
Asthma	Both	Yes		
CVD event	Both	Yes		
COPD	Both	Yes		
Diabetes requiring medication	Both	Yes		
CHF	Both	Yes		
Hepatic impairment	Both	Yes		
HIV	Both	Yes		
Hypertension requiring medication	Both	Yes		
Other immunosuppressive disorder	Both	Yes		
Malignancy (active or receiving treatment)	Both	Yes		
MI or other acute coronary syndrome	Both	Yes		
Renal impairment	Both	Yes		
CHF NYHA class (1-4)	TICO	No	Insufficienty data for validation	
Supp O2 before Covid	Both	Yes		
Flow rate before Covid	Both	Yes		
Renal replacement before Covid	Both	Yes		
Any SARS-CoV-2 vaccine?	Both	Yes	Included, but feature engineered to be compatible between TICO and ITAC datasets	
Remdesivir prior to randomization	Both	Yes		
Remdesivir at randomization	Both	No	Included, but feature engineered to be a composite feature with "Remdesivir prior to randomization".	
Remdesivir contraindicated	Both	Yes		
Remdesivir refused	Both	Yes		
Remdesivir other reason not infused	Both	Yes		
Respiratory rate (breaths per minute)	Both	Yes		
Oxygen saturation (%)	Both	Yes		
Measured on room air or supp O2	Both	Yes		
Temperature (celsius)	Both	Yes		
Temperature (fahrenheit)	Both	No	Only using metric units	

Systolic blood pressure (mmHg)	Both	Yes	
Diastolic blood pressure (mmHg)	Both	Yes	
Heart rate (bpm)	Both	Yes	
AVPU	Both	Yes	
Respiratory status at time of randomization	Both	Yes	
HFNC flow rate (liters/minute)	TICO	No	Insufficienty data for validation
HFNC FI02 (%)	TICO	No	Insufficienty data for validation
Conventional O2 flow rate (liters/minute)	Both	Yes	
Height	Both	Yes	
Weight	Both	Yes	
BMI	Both	Yes	
NEWS score	Both	Yes	
Taking any of the following meds?	Both	Yes	
Antibacterial	Both	Yes	
Intramuscular or IV antibacterial	Both	Yes	
Oral antibacterial	Both	Yes	
Antifungals	Both	Yes	
ACE inhibitor	Both	Yes	
ARBs	Both	Yes	
Antiplatelet/anticoagulant	Both	Yes	
Aspirin	Both	Yes	
Other antiplatelet agents	Both	Yes	
Heparin, proph dose	Both	Yes	
Heparin, inter dose	Both	Yes	
Heparin, therap dose	Both	Yes	
Warfarin	Both	Yes	
Direct oral anticoag (DOAC)	Both	Yes	
Antivirals against Covid	Both	Yes	
Favipiravir: label	Both	Yes	
Lopinavir/r	Both	Yes	
Other antiviral	Both	Yes	
Antirejection meds after transplant	Both	Yes	
Immune modulators	Both	Yes	
IL-1 inhibitor	Both	Yes	
IL-6 inhibitor	Both	Yes	
IFNs	Both	Yes	
JAK inhibitors	Both	Yes	
TNF inhibitors	Both	Yes	
Other IM	Both	Yes	
NSAIDS 7+ days	Both	Yes	
Corticosteroids (>10 mg prednisone):label	Both	Yes	
Biological meds to treat auto/cancer	Both	Yes	
Creatinine (mg/dl)	Both	Yes	
AST/SGOT (U/L) label	Both	Yes	
ALT/SGPT (U/L) label	Both	Yes	
White blood cell count	Both	Yes	
Haemoglobin (g/dl)	Both	Yes	
Platelets	Both	Yes	
Lymphocytes	Both	Yes	
CRP	Both	No	Only using CRP measures from central laboratory, and NOT measured at hospitals related to study site

Borg scale 0=nothing, 10=maximal	Both	Yes		86
**(CL) Lab test**				
Biorad S/C ratio day 0	Both	Yes		
Gen script binding inhibition (%) day 0	Both	Yes		
Gen script positive day 0	Both	Yes		
Biorad positive day 0	Both	Yes		
Quanterix antigen (ng/mL) day 0 (Plasma nucleocapsid)	Both	Yes		
Quanterix AG positive day 0	Both	Yes		
Quanterix antibody (ng/mL) day 0 (Anti spike)	Both	Yes		
Quanterix AB positive day 0	Both	Yes		
SARS Cov-2 RNA present	Both	Yes		
Delta variant	Both	Yes		
Delta category	Both	Yes		
Viral load copies/ml	Both	Yes		
RNA positive	Both	Yes		
D-Dimer	Both	Yes		
IL-6	Both	Yes		
CRP	Both	Yes		16
**Feature engineered variables**				
Infection period	Both	Yes	Used same definition as Aggarwal et al	
Number of comorbidities	Both	Yes	Used same definition as Aggarwal et al	
Geographical region	Both	Yes	Categorized according to continent	
Cardiovascular disease	Both	Yes		
Chronic kidney disease	Both	Yes		
Chronic lung disease	Both	Yes		
Immunocompromised state	Both	Yes		
Obesity	Both	Yes		8
Total features		117		
Total features without feature engineered variables		109		
Total included		107		
Percentage included		91,45299145		
Percentage of included that were feature engineered		7,476635514		
**Outcome features**				
Patient withdrew consent	Both	No	Used for censoring in the RSF model and for determining outcome=no in binary classification models	
Withdrawal time (days)	Both	No	Used for censoring in the RSF model and for determining outcome=no in binary classification models	
Death at 28 days	Both	Yes	Included only in by-proxy validation analysis	
Death at 90 days	TICO	Yes	Included only in primary development analysis	
Time-at-risk	Both	Yes	Included only in primary development analysis in the RSF model	
