# Supplementary material for: Comparing risk factors in severe COVID-19 using machine learning and non-machine learning methods: analysis from 2 international randomized controlled trials
Source: JAMIA Open. 2026 Jun 23;9(3):ooag079. doi: 10.1093/jamiaopen/ooag079 (PMC13289610; doi:10.1093/jamiaopen/ooag079)
Supplement: ooag079_Supplementary_Data [file ooag079_supplementary_data.zip › SupplementaryFile1.docx]

**Supplementary file 1**

**Tables**

| Supplementary Table 1 – baseline characteristics | | | | |
| --- | --- | --- | --- | --- |
| **Variables** | **TICO** | | **ITAC** | |
|  | n (%)/median (IQR) | Missing | n (%)/median (IQR) | Missing |
| **Total** | 2,625 | - | 579 |  |
| **Demographics** | | | | |
| **Sex**  Male  Female | 1,514 (57.7)  1,111 (42.3) | - | 329 (56.8)  250 (43.2) |  |
| **Age at randomization** | 57.0 (46.0-68.0) | - | 59.0 (49.0-70.0) |  |
| **Region**  Africa  North America  Asia  Europe  South America | 131 (5.0)  2,059 (78.4)  42 (1.6)  393 (15.0)  - | - | 41 (7.1)  248 (42.8)  53 (9.2)  233 (40.2)  4 (0.7) |  |
| **Ethnicity**  Asian  Black  Hispanic  White  Other | 121 (4.6)  629 (24.0)  486 (18.5)  1,515 (57.7)  101 (3.9) | - | 69 (11.9)  87 (15.0)  90 (15.5)  344 (59.4)  10 (1.7) |  |
| **Medical history** | | | | |
| **1 or more comorbidity**  Asthma  Cerebrovascular event  Chronic obstructive pulmonary disease  Diabetes  Congestive heart failure  Hepatic impairment  Human Immunodeficiency Virus (HIV)  Arterial hypertension  Immunosuppressive disorder (not HIV)  Malignancy  Acute coronary syndrome  Renal impairment | 1,690 (64.4)  260 (9.9)  37 (1.4)  167 (6.4)  740 (28.2)  116 (4.4)  40 (1.5)  42 (1.6)  1,201 (45.8)  82 (3.1)  106 (4.0)  45 (1.7)  260 (9.9) | - | 354 (61.0)  58 (10.0)  5 (0.9)  39 (6.7)  164 (28.3)  27 (4.7)  11 (1.9)  11 (1.9)  247 (42.7)  7 (1.2)  20 (3.5)  14 (2.4)  41 (7.1) |  |
| BMI (kg/m^2^) | 30.4 (26.3-36.0) | 9 (0.3) | 29.8 (25.8-34.7) | 5 (0.9) |
| **SARS-CoV-2 vaccinated?**  No  1 or more vaccinations | 2,098 (79.9)  527 (20.1) |  | 564 (97.9)  12 (2.1) |  |
| **Respiratory status** | | | | |
| **Borg scale**  Median  0-3  4-6  7-10 | 3.0 (1.0-4.0)  1,702 (64.8)  533 (20.3)  179 (6.8) | 211 (8.0) | 2.0 (0.5-3.0)  434 (74.9)  103 (17.8)  33 (5.7) | 9 (1.6) |
| **Pulmonary status**  Not receiving O_2_  Conventional oxygen therapy  High-flow nasal cannula  Non-invasive ventilation | 640 (24.4)  1699 (64.7)  258 (9.8)  28 (1.1) |  | 153 (26.4)  368 (63.6)  58 (10.0)  0 |  |
| **Medications** | | | | |
| **Treatment assigned**  Placebo  Active treatment | 1,147 (43.7)  1478 (56.3) | - | 284 (49.1)  295 (50.9) |  |
| Remdesivir before or at randomisation | 2,431 (92.6) |  | 549 (94.8) |  |
| **Antibacterial therapy**  Intramuscular or IV  Oral | 822 (31.3)  656 (25.0)  309 (11.8) |  | 244 (42.1)  183 (31.6)  118 (20.4) |  |
| Antifungals | 31 (1.2) |  | 3 (0.5) |  |
| ACE inhibitor | 272 (10.4) |  | 58 (10.0) |  |
| ARBs | 207 (7.9) |  | 68 (11.7) |  |
| **Antiplatelet/anticoagulant**  Aspirin  Other antiplatelet agents  Heparin prophylactic  Heparin intermediate  Heparin therapeutic  Warfarin  Direct oral anticoagulants  Antirejection meds post-transplant | 2,091 (79.7)  389 (14.8)  159 (6.1)  1,543 (58.8)  140 (5.3)  112 (4.3)  26 (1.0)  114 (4.3)  105 (4.0) |  | 429 (74.1)  62 (10.7)  47 (8.1)  295 (51.0)  44 (7.6)  13 (2.3)  2 (0.4)  26 (4.5)  2 (0.4) |  |
| Immune modulators  IL-1 inhibitor  IL-6 inhibitor  IFNs  JAK inhibitors  TNF inhibitors  Other | 169 (6.4)  1 (<0.1)  51 (1.9)  1 (<0.1)  87 (3.3)  2 (0.1)  29 (1.1) |  | 8 (1.4)  0  5 (0.9)  0  0  0  4 (0.7) |  |
| Non-steroidal anti- inflammatory drugs >7 days | 188 (7.2) |  | 44 (7.6) |  |
| Corticosteroids | 1,786 (68.0) |  | 327 (56.5) |  |
| Biological medications | 31 (1.2) |  | 3 (0.5) |  |
| **Peripheral blood samples** | | | | |
| Creatinine (mg/dl) | 0.9 (0.7-1.1) | 5 (0.2) | 0.8 (0.7-1.1) |  |
| Aspartate Aminotransferase (I/L) | 39.0 (27.0-57.0) | 2-38 (9.1) | 36.0 (25.0-52.0) | 108 (18.7) |
| Alanine Transaminase (U/L) | 33.0 (21.0-52.0) | 37 (1.4) | 33.0 (22.0-53.0) | 16 (2.8) |
| White blood count (x 10^6^/L cells/mm^3^) | 6.2 (4.4-8.7) | 5 (0.2) | 6.4 (4.7-9.3) |  |
| Haemoglobin (g/dl) | 13.2 (11.9-14.3) | 5 (0.2) | 13.2 (12.0-14.4) |  |
| Platelets (x 10^6^/L cells/mm^3^) | 209.0 (163.0-267.0) | 7 (0.3) | 218.0 (174.9-285.0) |  |
| Lymphocytes (x 10^6^/L cells/mm^3^) | 0.8 (0.6-1.2) | 46 (1.8) | 0.9 (0.7-1.4) | 8 (1.4) |
| C-reactive protein (mg/L) | 30.7 (14.5-56.1) | 270 (10.3) | 29.0 (11.0-57.7) | 12 (2.1) |
| Interleukin-6 (ng/L;pg/mL) | 5.8 (2.3-14.5) | 150 (5.7) | 5.3 (2.1-12.3) | 12 (2.1) |
| D-dimer (mg/L;ng/mL) | 0.9 (0.6-1.5) | 150 (5.7) | 866.7 (572.9-1,459.1) | 10 (1.7) |
| **Viral load, antibody- and antigen assays** | | | | |
| Upper respiratory tract quantified SARS-CoV-2 RNA (Viral load) copies/ml  Positive  Quantified | 2,189 (83.4)  16,125.0 (803.0-328,072.0) | 102 (3.9)  112 (4.3) | 446 (84.8)  25,611.0 (382.5-983,136.5) | 53 (9.2)  27 (4.7) |
| Antibody assays  GenScript (anti-S) binding inhibition (%)  Positive  Median  BioRad (anti-N) ratio  Positive  Median  Quanterix (anti-S)  Positive  Median | 1,312 (50.0)  32.1 (9.0-71.2)  1,586 (60.4)  3.0 (0.2-4.1)  1,164 (44.3)  648.5 (118.6-4,682.8) | 82 (3.1)  81 (3.1)  190 (7.2) | 275 (47.5)  27.1 (9.2-60.8)  391 (68.5)  3.5 (0.3-4.0)  23 (4.0)  652.5 (44.5-2,505.5) | 8 (1.4)  8 (1.4)  531 (91.7) |
| Antigen assay  Quanterix (N-antigen in plasma)  Positive  Median | 2,410 (91.8)  1,445.0 (234.0-4,731.0) | 82 (3.1) | 531 (93.2)  1,218.7 (171.0-4,095.0) | 9 (1.6) |
| **Mortality (Outcomes)** | | | | |
| 28-day mortality  Median time to death (28-day mortality)  90-day mortality  Median time to death  (90-day mortality) | 179 (6.8)  12 (8-18)  261 (9.9)  18 (10-31) | 27 (1.0)  41 (1.5) | 40 (6.9)  13 (7.5-19)  -  - | 1 (<1%) |

| Supplementary Table 2 - Mean performance measures of the best performing CatBoost model predicting 90-day mortality, applied to the test set. n =525, events =52. | |
| --- | --- |
| **Metric** |  |
| **PPV** | 0.42 |
| **NPV** | 0.96 |
| **Sensitivity** | 0.69 |
| **Specificity** | 0.90 |
| **Balanced Accuracy** | 0.79 |
| **MCC** | 0.48 |
| **ROCAUC** | 0.91 |
| **PRAUC** | 0.58 |
| **Brier Score** | 0.10 |
| **MRPAvg** | 0.66 |
| PPV = Positive predictive value; NPV = Negative predictive value; MCC = Matthew’s correlation coefficient; ROCAUC = Receiver operating characteristics area under the curve; PRAUC = Precision-recall area under the curve  MRPAvg = $\frac{MCC+ROCAUC+PRAUC}{3}$ | |

| Supplementary Table 3 - Performance measures derived from the transformed confusion matrix, derived from the Random Survival Forests analysis, predicting 90-day mortality, applied to the test set. | |
| --- | --- |
| **Metric** |  |
| **PPV** | 0.41 |
| **NPV** | 0.96 |
| **Sensitivity** | 0.62 |
| **Specificity** | 0.90 |
| **Balanced Accuracy** | 0.76 |
| **MCC** | 0.43 |
| PPV = Positive predictive value; NPV = Negative predictive value; MCC = Matthew’s correlation coefficient | |

| Supplementary Table 4 - Mean ± standard deviation of the performance measures of the five models predicting 28-day mortality, from 5-fold cross validation in the training set. n = 2100 | | | | | |
| --- | --- | --- | --- | --- | --- |
| **Metric** | **RandomForest** | **LightGBM** | **CatBoost** | **HistGBC** | **LogisticRegression** |
| **PPV** | 0.49 ± 0.19 | 0.32 ± 0.06 | 0.28 ± 0.07 | 0.25 ± 0.02 | 0.23 ± 0.03 |
| **NPV** | 0.95 ± 0.01 | 0.97 ± 0.01 | 0.97 ± 0.01 | 0.97 ± 0.0 | 0.97 ± 0.01 |
| **Sensitivity** | 0.23 ± 0.24 | 0.59 ± 0.14 | 0.59 ± 0.09 | 0.64 ± 0.06 | 0.7 ± 0.06 |
| **Specificity** | 0.97 ± 0.04 | 0.91 ± 0.01 | 0.88 ± 0.04 | 0.86 ± 0.02 | 0.82 ± 0.02 |
| **Balanced Accuracy** | 0.6 ± 0.1 | 0.75 ± 0.07 | 0.74 ± 0.05 | 0.75 ± 0.03 | 0.76 ± 0.04 |
| **MCC** | 0.26 ± 0.13 | 0.38 ± 0.09 | 0.34 ± 0.09 | 0.34 ± 0.03 | 0.32 ± 0.05 |
| **ROCAUC** | 0.88 ± 0.02 | 0.87 ± 0.03 | 0.86 ± 0.03 | 0.85 ± 0.04 | 0.83 ± 0.03 |
| **PRAUC** | 0.39 ± 0.08 | 0.39 ± 0.08 | 0.39 ± 0.11 | 0.31 ± 0.07 | 0.37 ± 0.09 |
| **Brier Score** | 0.07 ± 0.03 | 0.08 ± 0.01 | 0.11 ± 0.03 | 0.11 ± 0.01 | 0.13 ± 0.01 |
| PPV = Positive predictive value; NPV = Negative predictive value; MCC = Matthew’s correlation coefficient; ROCAUC = Receiver operating characteristics area under the curve; PRAUC = Precision-recall area under the curve | | | | | |

| Supplementary Table 5 - Mean performance measures of the best performing LightGBM model predicting 28-day mortality, applied to the test set. | |
| --- | --- |
| **Metric** |  |
| **PPV** | 0.32 |
| **NPV** | 0.97 |
| **Sensitivity** | 0.59 |
| **Specificity** | 0.91 |
| **Balanced Accuracy** | 0.75 |
| **MCC** | 0.38 |
| **ROCAUC** | 0.87 |
| **PRAUC** | 0.39 |
| **Brier Score** | 0.08 |
| **MRPAvg** | 0.55 |
| PPV = Positive predictive value; NPV = Negative predictive value; MCC = Matthew’s correlation coefficient; ROCAUC = Receiver operating characteristics area under the curve; PRAUC = Precision-recall area under the curve  MRPAvg = $\frac{MCC+ROCAUC+PRAUC}{3}$ | |

**Figures**

Supplementary Figure 1


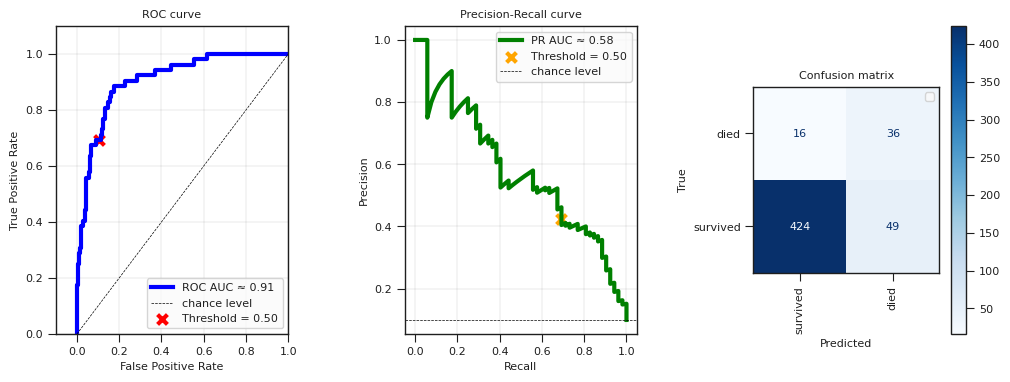


**Supplementary Figure 1 –** Receiver operating characteristics curve, Precision-Recall curve and confusion matrix derived from the CatBoost model applied to the test set.

Supplementary Figure 2


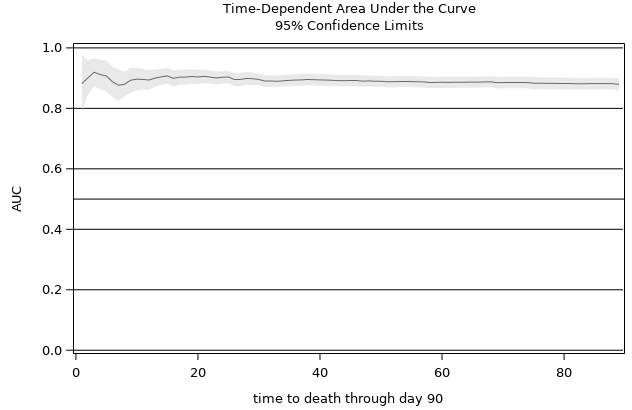


**Supplementary Figure 2 –** Integrated time-dependent AUC derived from the Cox model by Aggarwal et al, AUC = 0.90

Supplementary Figure 3


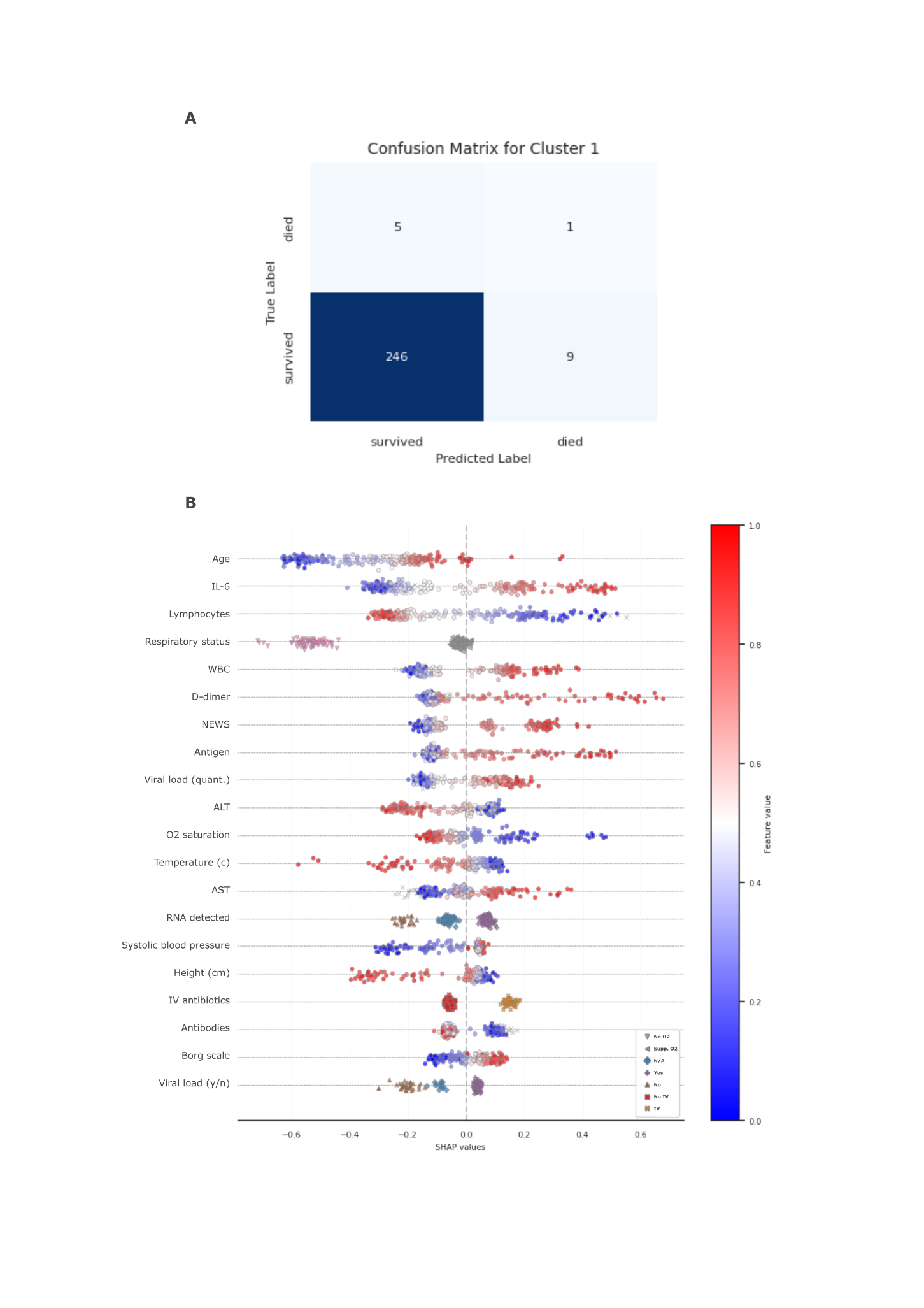


**Supplementary Figure 3 –** a) Confusion matrix and b) SHAP summary plot for a subset of patients from the test set. The cluster represents a low-risk subset of patients as reflected in the confusion matrix with the majority of patients who survived. SHAP clustering was performed using a hierarchical clustering method applied to SHAP values from all features and samples in the test set. The number of clusters was determined based on the Silhouette score, providing a data-driven approach to identify clustering. This method identifies patient subgroups with distinct SHAP value patterns, reflecting variations in model behavior. Feature values of continuous features correspond to the blue-to-red scale on the right. Feature values of categorical features are explained in the figure legend (yes/no/not applicable/unknown for binary features, specific categories for the multilevel “Respiratory Support” feature). IL-6 = interleukin-6; WBC = white blood cell count; NEWS = National Early Warning Score; ALT = alanine transaminase; AST = aspartate transaminase; RNA = Ribonucleic Acid; IV = intravenous; Supp. O2 = supplemental oxygen.

Supplementary Figure 4


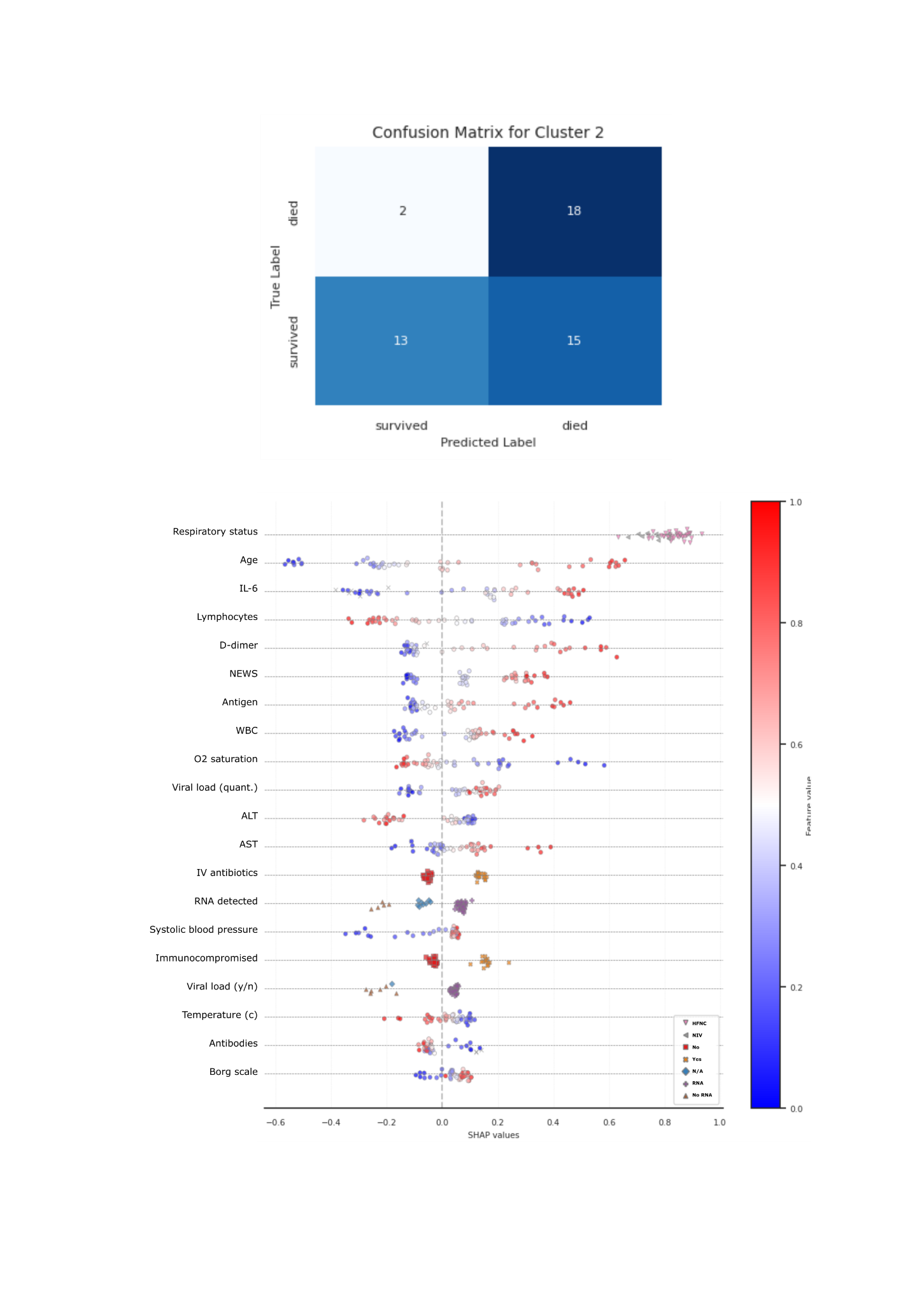


**Supplementary Figure 4 –** a) Confusion matrix and b) SHAP summary plot representing a high-risk patient subset from the test set as identified by the clustering of SHAP values. SHAP clustering was performed using a hierarchical clustering method applied to SHAP values from all features and samples in the test set. The number of clusters was determined based on the Silhouette score, providing a data-driven approach to identify clustering. This method identifies patient subgroups with distinct SHAP value patterns, reflecting variations in model behavior. Feature values of continuous features correspond to the blue-to-red scale on the right. Feature values of categorical features are explained in the figure legend (yes/no/not applicable/unknown for binary features, specific categories for the multilevel “Respiratory Support” feature). IL-6 = interleukin-6; WBC = white blood cell count; NEWS = National Early Warning Score; ALT = alanine transaminase; AST = aspartate transaminase; IV = intravenous; RNA = Ribonucleic Acid; HFNC = high-flow nasal cannula; NIV = Non-invasive ventilation

Supplementary Figure 5


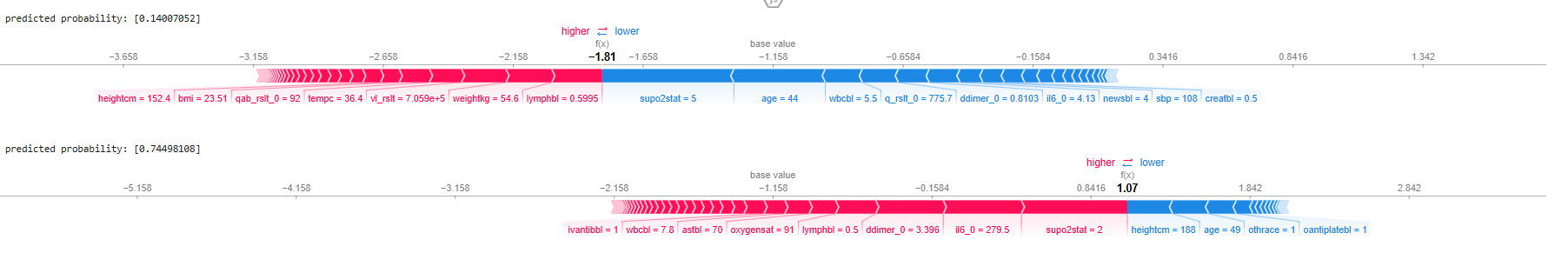


**Supplementary Figure 5 –** SHAP force plots for two individual patients. A SHAP force plot show which features and their respective value influence model prediction the most in a single observation.

Supplementary Figure 6


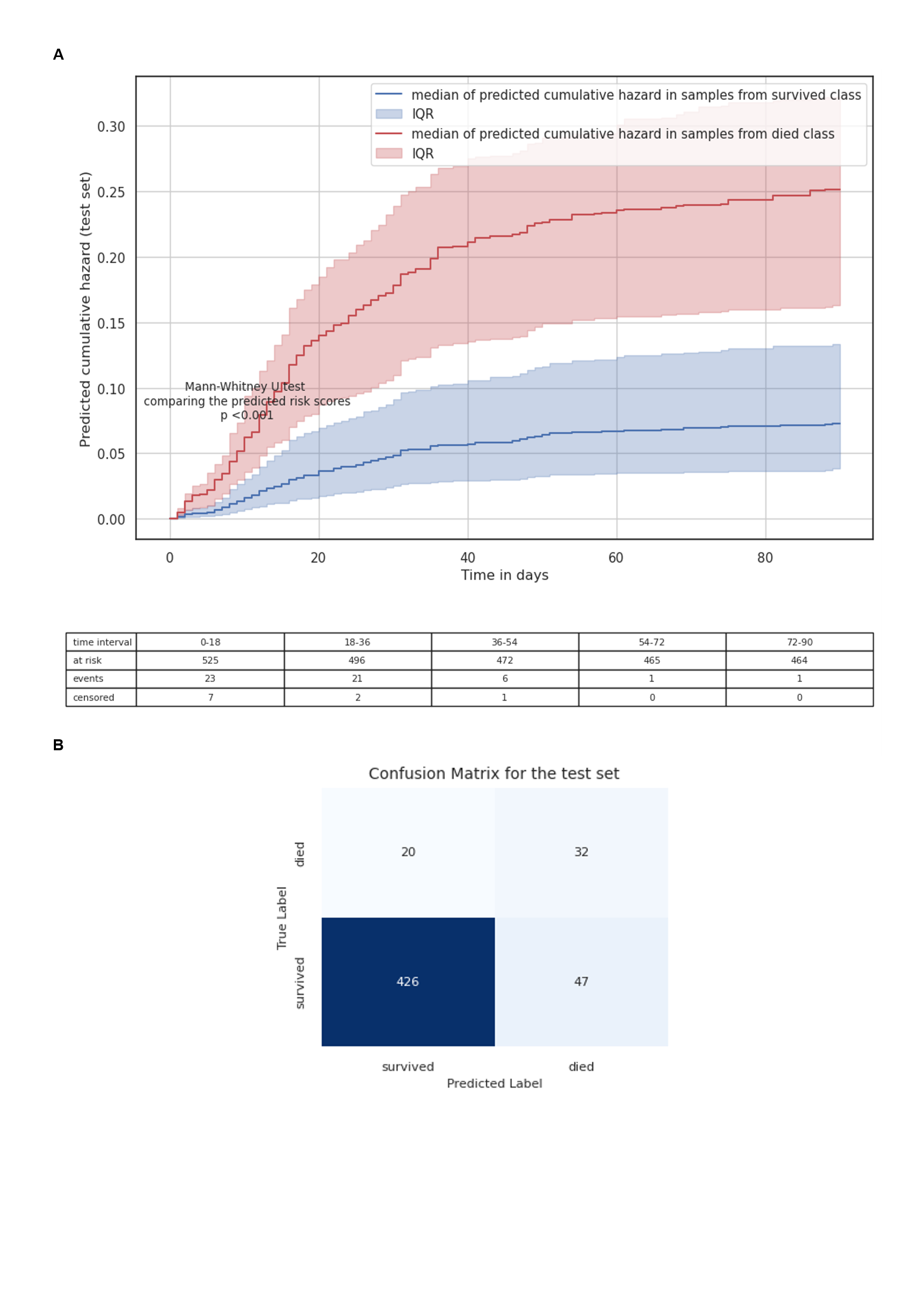


**Supplementary Figure 6 –** a) Predicted cumulative hazard plot derived from the Random Survival Forests analysis on the test set and b) Confusion Matrix transformed from the predicted cumulative hazards based on Euclidean distance of individual predicted cumulative hazards from the test set with the median of the predicted cumulative hazard for each class from the training set.

Supplementary Figure 7


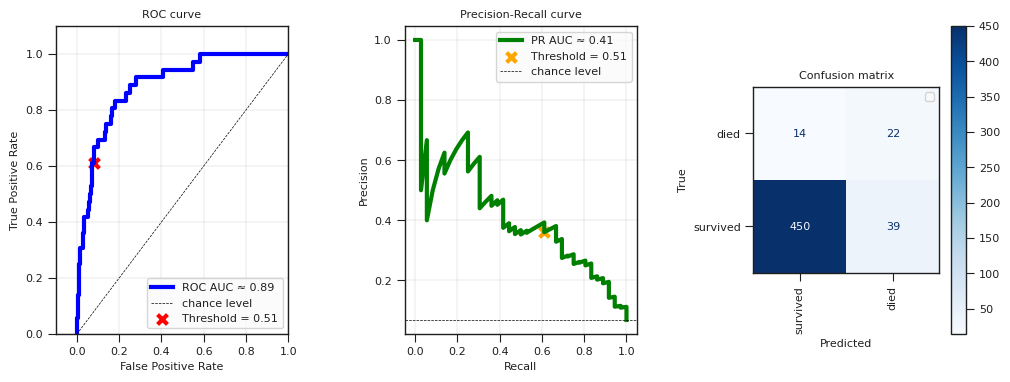


**Supplementary Figure 7 –** Receiver operating characteristics curve, Precision-Recall curve and confusion matrix derived from the LightGBM model applied to the test set.
